# Anesthetic management of a patient with left ventricular assist device undergoing robotic laparoscopic prostatectomy: a case report

**DOI:** 10.1186/s40981-020-00364-1

**Published:** 2020-07-25

**Authors:** Andrew J. B. Pisansky, Nelson Burbano-Vera, Matthias F. Stopfkuchen-Evans

**Affiliations:** 1 Department of Anesthesiology, Perioperative and Pain Medicine, Brigham and Women’s Hospital, Boston, MA USA; 2 Department of Anesthesiology and Pain Medicine, Cleveland Clinic Foundation, Cleveland, OH USA

**Keywords:** Left ventricular assist device, LVAD, Prostatectomy, Laparoscopic, Pneumoperitoneum, Trendelenberg

## Abstract

**Background:**

Patients with left ventricular assist devices (LVAD) require specific anesthetic and hemodynamic considerations. We report the specific anesthetic preparation and management in this scenario.

**Case presentation:**

We present the case of a 66-year-old male with a HeartMate II LVAD undergoing robotic prostatectomy for prostate cancer in the steep Trendelenburg position. We employed central venous and radial arterial access, LVAD pump parameters, near-infrared sensor of cerebral oximetry, and transesophageal echocardiography for monitoring. Hemodynamics were managed with nicardipine, dobutamine, epinephrine, and phenylephrine during abdominal insufflation, operative positioning, and desufflation. The patient had a successful procedure, was discharged on postoperative day 2, and achieved surgical cure of his prostate cancer.

**Discussion:**

By presenting the first detailed account of anesthetic management in this scenario, we provide a clinical vignette for use by the clinical anesthesiologist in his or her preparation prior to caring for this type of patient.

## Introduction

The use of left ventricular assist devices (LVADs) for patients with heart failure has become widespread. Although cardiac transplantation is considered definitive therapy, shortage of donor hearts has led to the increasing use of LVADs as a bridge to transplant or final therapy devices [1, 2]. Patients with these devices increasingly present for non-cardiac procedures and are cared for by both cardiac and non-cardiac anesthesiologists [3]. The ability to perform non-cardiac operations in LVAD patients has progressed from minor superficial procedures to open abdominal surgery and subsequently to laparoscopic abdominal surgery [4–8]. The majority (> 95%) of LVADs implanted since 2010 are continuous flow devices, which create special hemodynamic considerations related to their sensitivity to preload and afterload conditions [9]. Potential adverse events in LVAD patients during anesthetic administration included suction events (i.e., LVAD flow exceeding available LV preload) which may precipitate ventricular arrythmia; however, there has been limited attention paid to the strategies of anesthetic and hemodynamic management employed in the care of these patients. We describe a case in which a patient with an LVAD underwent a robotic prostatectomy in the steep Trendelenburg position for treatment of prostate cancer and provide management detail not previously present in the literature. HIPAA authorization has been obtained from the patient.

## Case description

A 66-year-old male patient with a HeartMate II model LVAD (Abbott Laboratories, Abbott Park, IL) 4 months postoperative from implant was planned for robotic prostatectomy for prostate cancer after multidisciplinary oncologic case discussion, with a goal for a surgical cure in order to obtain candidacy for heart transplantation. His past medical history included viral cardiomyopathy with severe left ventricular dysfunction (EF 20%), ventricular tachycardia status post-implanted cardiac defibrillator (ICD), stage 2 chronic kidney disease, and hypertension.

Preoperative evaluation with a ramp transthoracic echocardiogram (TTE) demonstrated an optimal pump speed of 5300 revolutions per minute (RPM). This type of TTE is a protocolized assessment of LVAD operation during which continuous evaluation of the heart is used to determine an optimal RPM at which the patient’s ventricular function, aortic opening, and cardiac output are optimal [10]. The TTE showed minimal left ventricular contraction, mild aortic insufficiency with preserved valve opening approximately every 2–3 beats, moderate right ventricular dysfunction, mild tricuspid regurgitation, and no mitral regurgitation. He was admitted to the hospital the night prior to the procedure for LVAD interrogation, which showed flow rate 4.2 L/min, speed 5300 RPM, pulsatility index (PI) 4.5, and power 3.9 W. Pulsatility index is a dimensionless measure of left ventricle (LV) contribution to cardiac output as it describes how pulsatile flow through the pump is at a given time. PI is dependent on the relationship between the pressure generated by the LV and the pressure generated by the LVAD. In a state with low pulsatility index, the left ventricle does not generate a pressure sufficient to exceed aortic root pressure and therefore results in the aortic valve not opening during systole with low PI. ICD interrogation showed no recent VT events, VT threshold at 170 beats per minute with anti-tachycardia pacing available, 10 years battery life, and less than 0.1% ventricular pacing. Anticoagulation was managed with warfarin, physical exam was unremarkable given his past medical history, and electrocardiogram showed normal sinus rhythm.

Warfarin was held the night prior to the surgery. On the morning of prostatectomy, prothrombin international normalized ratio (INR) resulted as 3.2. He was given prothrombin complex concentrate (PCC; CSL Behring, King of Prussia, PA). PT-INR was again drawn upon arrival in the operating room and resulted at 1.4.

The intraoperative team included the urologic surgeon, general anesthesiologist, pharmacist, and a cardiac perfusionist. After placement of ASA standard monitors, a 20-gage right radial arterial line was placed under ultrasound guidance. Central venous access was obtained via the right internal jugular vein. Additional variables monitored included near-infrared cerebral oximetry (INVOS, Medtronic Inc., Minneapolis, MN), bispectral index monitor (Medtronic Inc, Minneapolis, MN), and LVAD power index and rotation speed communicated to the anesthesia team by the cardiac perfusionist.

**Fig. 1:**
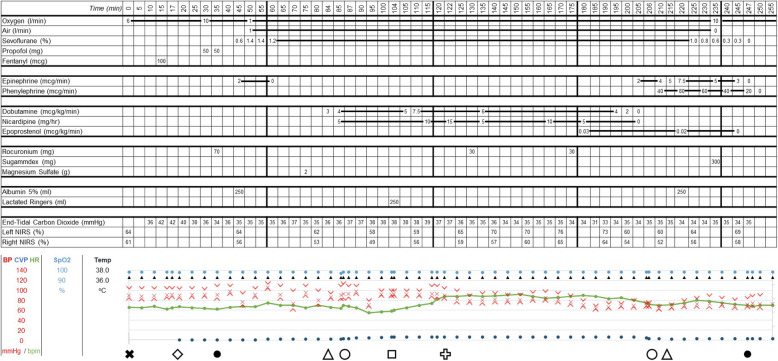
Anesthetic record. black X, arterial line placement; white diamond, central line placement; black circle, tracheal intubation and extubation; white triangle, abdominal insufflation and desufflation; white circle into and out of steep Trendelenberg position; white square, aortic valve opening; white cross, transesophageal echocardiogram (TEE). BP, blood pressure; CVP, central venous pressure; HR, heart rate; SpO_2_, oxygen saturation percentage; Temp, temperature

Anesthesia was induced using fentanyl 1.5 mcg/kg, propofol 1 mg/kg, and rocuronium 1.2 mg/kg, which resulted in relative hypotension that responded to a bolus of 250 ml of 5% albumin. Rapid sequence induction was chosen to rapidly secure the airway and reach a stable plane of anesthesia using a volatile anesthetic (sevoflurane). Albumin was chosen as it is our institutional colloid of choice. Full details of the anesthetic record are provided in Fig. 1.

Surgical insufflation of the abdomen resulted in an acute rise in blood pressure, which was associated with the decrease in LVAD flow rate and increased power requirement presumed due to high afterload. This was treated with 0.75 mg/kg bolus of propofol and initiation of dobutamine and nicardipine infusions with good effect. The patient was placed in a Trendelenburg position in a stepwise manner while monitoring hemodynamics and LVAD indices. Trendelenburg positioning was associated with a rise in the central venous pressure (CVP), though this was tolerated. The LVAD showed the following parameters: pulsatility index 3.9, flow 4.5 L/min, rpm 5300, and power 3.8 W.

**Fig. 2:**
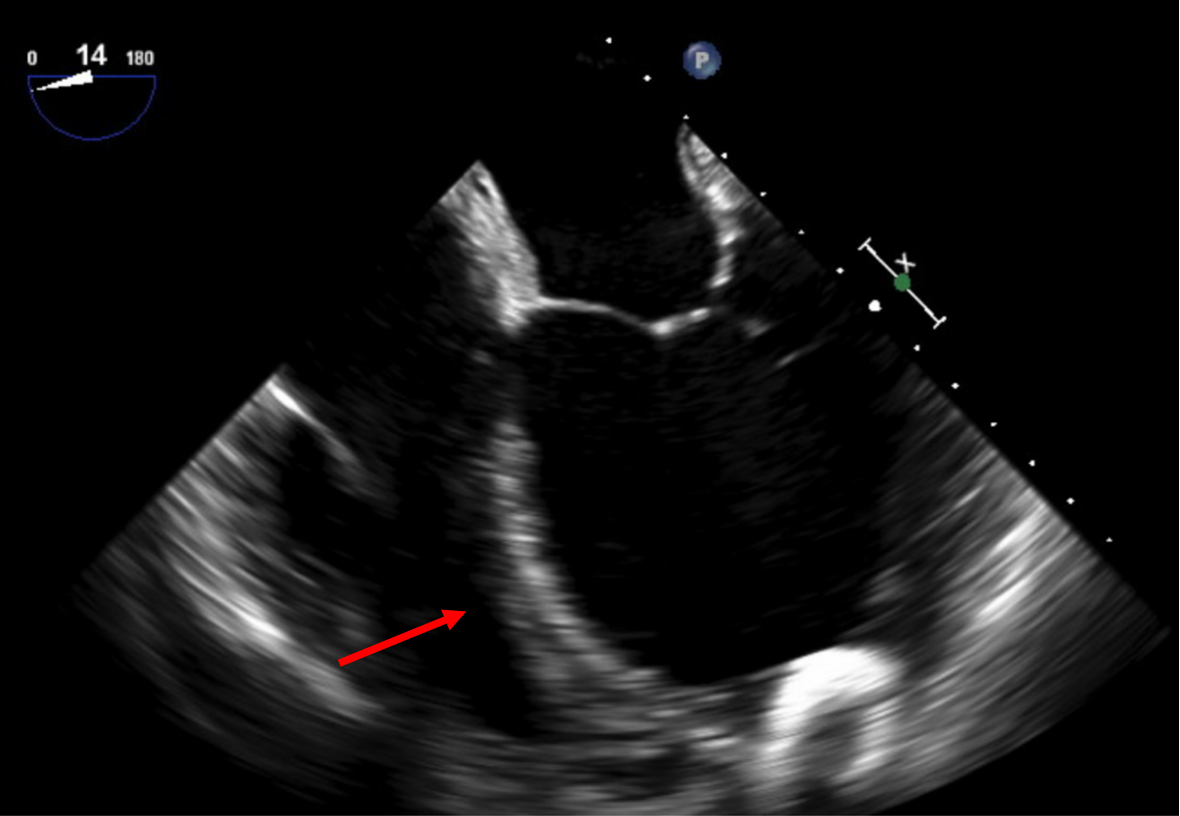
Transesophageal echocardiogram image showing bowing of the intraventricular septum bulging to the right during diastole. Arrow indicates intraventricular septum bowing

Approximately midway through the procedure, the pulsatility index began to decrease and central venous pressure (CVP) increased by 50%. This generated concern for worsening right ventricular function creating decreased left ventricular and LVAD preload. Cardiac anesthesia was called for intraoperative transesophageal echocardiogram (TEE). TEE demonstrated mild bowing of the intraventricular septum into the right ventricle and moderate right ventricular dysfunction (see Fig. 2). Inhaled epoprostenol was initiated to further optimize the right ventricular function. Vasoactive medications (nicardipine; dobutamine) were continued. The LVAD pulsatility index, flow, and RPM returned to their prior values (Table 1).

**Table 1:** Hemodynamic parameters and patient positioning changes

Chart time (minutes since case start)	Event	LVAD PI	LVAD flow (L/min)
84	Abdominal insufflation	N/A	N/A
87	Trendelenberg positioning	5.9	3.8
104	Aortic valve opening	2.7	4.5
122	TEE insertion	2.8	4.5
125	None	3.3	4.4
175	None	2.6	4.5
206	Abdominal desufflation	N/A	N/A
210	Patient out of Trendelenberg	2.1	4.5

*PI* pulsatility index, *LVAD* left ventricular assist device, *TEE* transesophageal echocardiogram, *Chart time* timestamp listed on anesthetic record provided in Fig. 1

Robotic prostatectomy was completed in the standard fashion. Ten minutes prior to abdominal desufflation, nicardipine infusion was paused and dobutamine infusion rate was reduced. At the time of desufflation, there was an acute drop in arterial blood pressure which required initiation of phenylephrine and epinephrine infusions as well as a crystalloid bolus of 250 ml. Neuromuscular blockade was reversed using sugammadex (Merck, Kenilworth, NJ), and the patient was extubated. All vasoactive medications were able to be discontinued at this time, and the patient was transported to the cardiac surgery intermediate care unit in stable condition.

Postoperatively, he did not require vasoactive infusions. PT-INR was drawn postoperatively at 5 h after administration of prothrombin concentrate and resulted at 1.5. His preoperative dose of warfarin was resumed on the evening of postoperative day 0, and INR on postoperative day 1 was 2.0. The remainder of his postoperative laboratory studies was stable from preoperative values. He was discharged home on postoperative day 2. Subsequent evaluation by urologic surgery has determined that the procedure was curative. He is currently undergoing evaluation for cardiac transplant.

## Discussion

In this case report, we share our experience with the anesthetic management of a patient with an LVAD undergoing laparoscopic abdominal surgery in steep Trendelenburg positioning.

The ramp TTE is perhaps the most important preoperative test in this situation and was initially reported as a means to optimize LVAD pump speed and to diagnose device thrombosis [10]. The test is diagnostic for LVAD pump function but may also contribute to preoperative optimization of the pump in order to reduce the likelihood of left ventricular collapse related to pump volume extraction exceeding available LV preload under baseline loading conditions. Interpretation of the ramp TTE has been reviewed elsewhere, with the key consideration being the intermittent or complete lack of pulsatile blood flow [11].

The hemodynamics of patients with LVAD devices have unique considerations with respect to cardiac output [11]. Blood flow through the device is determined by the pressure gradient across the pump and the pump speed. Increased pressure gradient across the pump will decrease flow, which creates an inverse relationship between mean arterial pressure (MAP) and pump flow rate. Additionally, the right ventricle must deliver adequate preload to prevent left ventricular collapse due to excess extraction of blood volume by the pump (i.e., “suction event”). Both preload and afterload are affected by Trendelenburg positioning and abdominal insufflation. Measurement of cardiac parameters in healthy patients has demonstrated that while Trendelenburg position increases stroke volume, the addition of pneumoperitoneum also increases MAP and systemic vascular resistance (SVR), both of which have implications for LVAD function [12]. While Trendelenburg position may favor loading characteristics for the LVAD, the increased afterload associated with pneumoperitoneum likely decreases pump flow. In this case report, we describe our experience with managing these conditions, including the need for afterload reduction during abdominal insufflation and preload management during induction of anesthesia and abdominal desufflation.

The literature does not provide specific recommendations for perioperative management of anticoagulation in LVAD patients. The majority of literature focuses on institutional experience and case reports, though it appears that the majority of patients with LVAD presenting for non-cardiac surgery receive some type of pharmacologic reversal [13].

Our case supports previous findings that patients with LVAD devices may safely tolerate procedures that require abdominal insufflation as well as potentially problematic positioning such as steep Trendelenburg. This was demonstrated by Khemees et al., although that report and others for laparoscopic nephrectomy [8] and sleeve gastrectomy [14] focus primarily on the surgical management and provide limited information regarding the anesthetic considerations and techniques employed [15].

As the number of patients with ventricular assist devices presenting for non-cardiac procedures grows, the economics and workforce supply considerations of who manages these patients may favor generalist responsibility with specialist consultation as needed. In our case, preoperative evaluation and optimization facilitated this strategy. If a combination of system-based approaches, local expertise, and interdisciplinary collaboration can be established, the value proposition of managing these patients using a generalist model may create substantial savings for healthcare systems that care for these patients.

In this report, we present a case of moderately complex non-cardiac surgery delivered by one potential configuration of the care team with a specific focus on anesthetic management and considerations. General anesthesiologists with adequate motivation, proper preparation, and collaborative systems may be able to provide safe anesthetic care and positive outcomes for LVAD patients undergoing non-cardiac surgery.

## Data Availability

The source material is available upon request.
